# Experience on how to implement a preanalytical and POCT unit in Madrid’s IFEMA field hospital during this unprecedented COVID-19 emergency

**DOI:** 10.11613/BM.2020.030403

**Published:** 2020-10-15

**Authors:** Jorge Díaz–Garzón, Paloma Oliver, Gema Crespo, Marta Duque, Pilar Fernandez-Calle, Marta Gómez, Roberto Mora, Isabel Moreno, Lydia Pascual, Ana-Laila Qasem, Olaia Rodriguez-Fraga, Manuela Simón, Antonio Buño

**Affiliations:** Laboratory Medicine Department, La Paz University Hospital, Madrid, Spain

**Keywords:** COVID-19, field hospital, laboratory organization and management, preanalytics, POCT

## Abstract

To fight the virus SARS-CoV-2 spread to Europe from China and to give support to the collapsed public health system, the Spanish Health Authorities developed a field hospital located in the facilities of Madrid exhibition centre (IFEMA) to admit and treat patients diagnosed with SARS-CoV-2 infectious disease (COVID-19). The Department of Laboratory Medicine of La Paz University Hospital in Madrid (LMD-HULP) was designated to provide laboratory services. Due to the emergency, the IFEMA field hospital had to be prepared for patient admission in less than 1 week and the laboratory professionals had to collaborate in a multidisciplinary group to assure that resources were available to start on time. The LMD-HULP participated together with the managers in the design of the tests portfolio and the integration of the healthcare information systems (IS) (hospital IS, laboratory IS and POCT management system). Laboratorians developed a strategy to quickly train clinicians and nurses on test requests, sample collection procedures and management/handling of the POCT blood gas analyser both by written materials and training videos. The IFEMA´s preanalytical unit managed 3782 requests, and more than 11,000 samples from March 27th to April 30th. Furthermore, 1151 samples were measured by blood gas analysers. In conclusion, laboratory professionals must be resilient and have to respond timely in emergencies as this pandemic. The lab’s personnel selection, design and monitoring indicators to maintain and further improve the quality and value of laboratory services is crucial to support medical decision making and provide better patient care.

The pandemic caused by the severe acute respiratory syndrome corona virus 2 (SARS-CoV-2) in late 2019 was firstly described in China and spread to Europe triggering an emergency alert. To fight the virus and to give support to the collapsed public health system, the Spanish Health Authorities developed a field hospital to admit and treat patients diagnosed with SARS-CoV-2 infectious disease (COVID-19). The hospital was located in the facilities of Madrid exhibition centre (IFEMA). The Department of Laboratory Medicine of La Paz University Hospital in Madrid (LMD-HULP) was designated to provide laboratory services. For this purpose, the LMD-HULP appointed professionals in charge of designing and supervising the implementation and work-flows of preanalytical, analytical and point-of-care testing (POCT) procedures. In total, a group of 7 laboratory technicians, 4 residents and 7 specialists in laboratory medicine coordinated and assisted both preanalytical and POCT units, and alternatively rendered service. The number of professionals working at the same time and the time-frame was modulated depending on the needs.

The field hospital was initially made up of one pavilion (500 beds) and then it was expanded to two pavilions with more complex resources to cover the patient’s needs with a total capacity of 1350 hospital beds (96 in intensive care unit) and 24 nursing controls.

Due to the emergency, the IFEMA field hospital had to be prepared for patient admission in less than 1 week, including all the infrastructures in empty pavilions. Then, laboratory facilities had to be implemented in record time, counting with the collaboration of soldiers and voluntary fire-fighters to carry the furniture and setting the preanalytical area in less than 24 hours. Laboratory professionals also had to coordinate and collaborate in a multidisciplinary group (informaticians, warehouse operators, administrative, nursing coordinator, directive board, *etc.*) to assure that the sample collection material, POCT analysers, reagents and other material resources were available to start on time as well as an appropriate warehouse had to be organized. And last but not least before starting, the LMD-HULP participated together with the managers in the design of the integration of the healthcare information systems (IS) (hospital IS (HIS), laboratory IS (LIS), POCT management system). A lot of time and human resources were invested in this task and hundreds of e-mails and phone calls were made to achieve the integration of the IS in less than 1 week. That was made to be fully connected to the HIS and making data easily accessible by all parties to ensure an efficient process. It is well known that an investment in a correct and robust infrastructure in the IS is time-worthy and impacts directly on patient safety because frequent human errors can be avoided. This integration guaranteed electronic test requesting, blood collection and the availability of patient results in both routine and emergency contexts, and at the same time allowed traceability from clinician’s orders to the final results report.

Laboratorians agreed on the tests portfolio with the medical directors, including three different profiles categorized by disease severity, supporting the diagnosis, treatment and the specific clinical needs of COVID-19. The HIS configuration was readopted to print laboratory request forms with sample collection instructions and barcode labels to properly draw and identify the samples, avoiding misidentification and preanalytical errors. Numerous visits to the 24 nursing units were done to train and qualify the staff, and a work instruction on how to print the laboratory request forms was made and uploaded to a shared folder available in all computers to be consulted.

Regarding the preanalytical procedures, beside the nursing training to sample collection procedures, specific procedures for test requests and blood collection, both by written materials and training videos, were designed. Laboratorians developed a strategy to quickly train clinicians and nurses in these tasks and also in the management/handling of the POCT blood gas analysers.

With respect to quality assurance, the laboratory established essential preanalytical quality indicators to monitor both units and detect any incidences that could impact on patient care, to carry out timely actions and prevent errors.

The workforce of the field hospital was made up of professionals from many different centres and volunteers, with a varied level of expertise. This fact reinforced the crucial role of using educational strategies and training procedures that were particularly difficult in such a challenging environment. Another basic task was the training on the use of the personal protective equipment (PPI) that for sure made every single step even more difficult.

Once samples were collected, the nursing units sent them to the preanalytical area where they were centralized, reviewed and properly prepared to be sent in certified sample transport boxes to the LMD-HULP. A daily transport route was set three times (9.00, 11.00 and 14.00) and the STAT samples were sent through extra emergency transport at any time.

In the same way, the POCT unit set several meetings to qualify the professionals. Before performing the blood gas analysis, samples were identified using an unequivocal number (hospital patient ID). In total, 9 blood gas analysers (8 ABL90 Flex and 1 ABL80; Radiometer, Brønshøj, Denmark), which were previously verified by the laboratory to assure accurate results, were distributed throughout both pavilions. These analysers were connected to the LMD-HULP´s POCT data management system (Aqure point-of-care IT solution, Radiometer, Brønshøj, Denmark) and continuously monitored (24h).

The IFEMA´s preanalytical unit managed 3782 requests, and more than 11,000 samples were routed to the emergency, microbiology and pharmacology laboratories from March 27th to April 30th. Furthermore, 1151 samples were measured by blood gas analysers. Due to the LIS integration, all the results were integrated into the patient medical record, closing the entire cycle of the total testing process from the pre-pre-analytical until the post-post-analytical phase with the quality assurance level that only laboratorians can provide.

In conclusion, laboratory professionals must be resilient and have to respond timely in emergencies as this pandemic. The lab’s personnel selection, design (request profiles, work instructions, integration of IS, samples transportation) and monitoring indicators to maintain and further improve the quality and value of laboratory services is crucial to support medical decision making and provide better patient care.

